# Implementing a digital treatment program for patients with irritable bowel syndrome into routine care: a qualitative evaluation of barriers and facilitators perceived by key stakeholders

**DOI:** 10.1186/s12913-025-13171-0

**Published:** 2025-08-09

**Authors:** Camilla Thuen, Hanne Karoline Hinderaker, Elisabeth Kjelsvik Steinsvik, Linn Anja Slåke Vikøren, Gülen Arslan Lied, Birgitte Berentsen, Robin Maria Francisca Kenter

**Affiliations:** 1 Center for Nutrition, Department of Clinical Medicine, University of Bergen, Bergen, Norway; 2 Research Centre for digital mental health services, Division of Psychiatry, Haukeland University Hospital, Bergen, Norway; 3 Department of Clinical Medicine, University of Bergen, Bergen, Norway; 4 Department of Medicine, Section of Gastroenterology, Haukeland University Hospital, Bergen, Norway; 5 Department of Medicine, Section of Clinical Nutrition, Haukeland University Hospital, Bergen, Norway

**Keywords:** Irritable bowel syndrome, Functional Gastrointestinal disorders (FGID), Disturbances of brain gut interaction (DBGI), Digital somatic treatment, Digital treatment program, EHealth, The consolidated framework for implementation research (CFIR), Barriers, Facilitators, Qualitative study

## Abstract

**Background:**

Digital treatment programs for irritable bowel syndrome offer multiple advantages as they enhance patient access to effective multidisciplinary lifestyle interventions while reducing individual symptom burden, in addition to alleviating strain on the healthcare systems. Despite the substantial benefits offered by digital treatment programs, exploring their implementation into routine care remain insufficient. This study aimed to systematically identify factors influencing the implementation of a digital treatment program for patients with irritable bowel syndrome as a routine intervention within a somatic secondary healthcare system.

**Methods:**

A qualitative study design was employed, and data was collected through semi-structured interviews with 16 key stakeholders involved in the implementation process. The sample represented several roles, ranging from high level leaders, implementation leads and physicians referring patients to the program. The Consolidated Framework for Implementation Research was utilised to guide the development of the interview guide, data collection, data analysis and interpretation of the results.

**Results:**

Key barriers were identified in the Outer Setting Domain: *financing* and Implementation Process Domain: *planning*, whereas key facilitators were found in the Innovation Domain: *innovation source* and *innovation relative advantage*; Outer Setting Domain: *critical incidents*; Inner Setting Domain: *tension for change;* and Implementation Process Domain: *teaming*. Among the individuals involved in the implementation, three key characteristics were identified as facilitating the implementation process: *capability* among high-level leaders, *opportunity* among mid-level leaders and referring physicians, in addition to *motivation* among deliverers, implementation leads, and other implementation support.

**Conclusion:**

This study provides novel insight into the multilayered factors that influence the implementation process of a digital treatment program in routine care of patients with irritable bowel syndrome. Our findings demonstrate that leveraging factors that aid implementation is important, while addressing barriers is crucial to ensure sustainable implementation. Moreover, our findings provide valuable insights for future digital healthcare initiatives, highlighting the value of early planning, engagement of stakeholders, and recognition of the dynamic nature of factors influencing the implementation process in secondary healthcare systems. Secondary healthcare systems should prioritise early strategic planning, identify and engage diverse stakeholders, and develop adaptive approaches that respond to the dynamic interplay of implementation factors. We suggest that this may facilitate the adoption of effective digital interventions for chronic conditions, also beyond irritable bowel syndrome.

**Trial registration:**

The study fell outside the scope of the Health Research Act (1) and could be conducted without the approval of the regional ethics committee. The study was registered in Helse Bergen’s internal control system for research, innovation and quality improvement with project number 5226-5226 and received approval from the local Data Protection Officer who reviewed the study and the materials.

**Supplementary Information:**

The online version contains supplementary material available at 10.1186/s12913-025-13171-0.

## Introduction

Irritable bowel syndrome (IBS) is a chronic condition characterised by recurrent abdominal pain and alterations in stool form or frequency [2]. IBS is associated with reduced quality of life and impaired work ability and is estimated to affect between 4% and 9% of individuals globally, with Norwegian data suggesting a prevalence of 10% [3, 4]. There is currently no effective cure for IBS, but several effective lifestyle interventions exist [5]. Owing to a lack of trained professionals, travel distances and costs, access to treatment options for patients with IBS is often limited. To address these challenges, the National Competence Centre for Functional Gastrointestinal Diseases in Bergen, Norway, initiated the development of a digital multidisciplinary self-care management programme for patients with IBS in 2016. This digital treatment program was based on an existing onsite multidisciplinary group-based education program, which was established and well-regarded by patients and health professionals [6]. By offering education alongside asynchronous follow-up by a clinical dietitian, the digital treatment program ensures that patients receive personalised guidance and practical tools to cope with their symptoms without the constraints of scheduling and travel. While the digital treatment program started as a research project, it is now implemented as a regional treatment option for patients with IBS in the Western Norway Regional Health Authority (RHA). In Norway, this is the first digital treatment program in the somatic healthcare system that aimed at being implemented in routine care nationwide. The digital treatment program is described in detail elsewhere [6]. The present study was conducted ten months after the initial implementation of the digital treatment program in the Western Norway RHA with the aim of systematically exploring the barriers and facilitators in the implementation process using the Consolidated Framework for Implementation Research (CFIR) as a guiding framework [7]. By identifying key factors influencing the implementation process, our findings may offer practical insights that can enhance the ongoing rollout of the program and support the successful implementation of similar asynchronous digital treatment programs.

## Methods

### Study design

The implementation of the digital treatment program for IBS was evaluated using a qualitative design, with data collected through semi-structured interviews with key stakeholders. Stakeholders were identified in advance through a strategic sampling method with the aim of representing a range of perspectives related to the implementation process, ranging from high level leaders to physicians referring patients to the program. Stakeholders were invited based on their area of expertise (e.g. physicians with a focus on gastroenterology and therefore had many patients with IBS) or direct involvement in the implementation process. Additionally, the snowball sampling method was employed, and three additional participants were recruited. All stakeholders were invited to participate via email. If they did not respond to the first email, a follow-up email was sent. If there was still no response, no further reminders were sent. The interview guide was developed with guidance from the CFIR (additional file 1). The CFIR is one of the most used frameworks in implementation research for explaining evidence-based factors that influence implementation efforts [7]. The CFIR is organised into five domains: (1) characteristics of the innovation (i.e. the digital treatment program), (2) factors in the outer setting (outside the predefined implementation site, i.e. outside the Western Norwegian RHA), (3) factors in the inner setting (inside the predefined implementation site, i.e. inside the Western Norwegian RHA), (4) roles and characteristics of individuals involved in the implementation, and (5) characteristics of the implementation process. Under these five domains, 48 constructs and 19 subconstructs aim to contextualise the information [7]. We evaluated all constructs and subconstructs. Constructs that were not mentioned by the participants were still assessed for their relevance, as their absence could indicate potential barriers or facilitators. In this article, we only report on the constructs identified as relevant during the analysis by three members of the research team (CT, HKH and RKFM). The interviews were conducted in October 2024. One interview was conducted on Microsoft Teams, while the other interviews were conducted at the participant’s workplace. A group interview was conducted with two intervention deliverers to encourage dynamic interaction and shared reflection on their joint experience and accommodate the scheduling needs of this specific team of participants who worked closely together. Due to the snowball sampling method, one of the participants who worked closely with the deliverers as implementation support also participated in the group interview. Given their roles and close collaboration this format was considered appropriate. The same semi-structured interview guide was used to maintain consistency across interviews. All other interviews were conducted individually. This approach was chosen to accommodate the participants’ schedules and geographical locations, and to protect confidentiality. Individual interview also provided a setting in which participants could speak freely about potential sensitive topics related to the organisational processes and implementation challenges. All interviews were recorded with an audio recorder (Olympus WS-853) and were conducted by the same two members of the research team (CT and HKH), both present during each interview. Field notes were taken by both interviewers and these notes were used to support and contextualize the analysis. The completed Standards for Reporting Qualitative Research (SRQR) checklist [8] is included as additional file 2.

### Study setting

The study setting provides an important context for understanding the barriers and facilitators identified in our study. The implementation of Norway’s first nationwide digital treatment program was located at Haukeland University Hospital (HUH) in Bergen, which houses the National Centre for Functional Gastrointestinal Diseases. The National Centre received governmental earmarked funding through the Norwegian Directorate between 2014 and 2024. While the National Centre developed the program, operational responsibility transitioned to the Department of Medicine for routine care implementation in the autumn of 2023, with three collaborating sections: Clinical Nutrition (program delivery and day-to-day digital treatment), Gastroenterology (referrals and medical oversight), and Administrative Services (administrative support). Section for eHealth, HUH, was and currently is the digital treatment platform’s system administrator and operations manager. Marketing and further implementation is still under the responsibility of the National Centre. Being responsible for handling the referrals, the Section of Gastroenterology receives referrals from gastroenterologists working at hospitals in the Western Norway RHA, in addition to private consulting gastroenterologists and general practitioners. The clinical dietitians from the Section of Clinical Nutrition carry out the initial group video session, where they introduce the patients to the digital treatment program. After the video session, the secretary from the Section of Administrative Services and the clinical dietitians collaborates on the administrative part of the patient flow, whereas the clinical dietitians solely handle the clinical part of the patient flow. All the clinical dietitians involved also work face-to-face with patients. Comprehensive health services are provided to all Norwegian residents, ensuring universal health coverage [9]. While the out-of pocket payments (OPPs) are moderate, public funding accounts for 85.8% of Norway’s total healthcare expenditure, which represents the highest proportion in Europe [9]. The Norwegian secondary healthcare system is divided into four RHAs, each funded through a combination of a basic fixed grant determined by demographic and regional factors of the catchment area, and a performance-based grant (ISF; Norwegian abbreviation for performance-based grant), where hospitals receive funding based on the volume and type of treatments provided [10]. Treatments within the ISF-system are categorised using the Diagnosis-Related Group (DRG) system, which organises hospital stays based on medical and administrative data [10]. The classification relies on the International Classification of Diseases (ICD-10) for diagnoses and the Nordic Classification of Medical Procedures (NCMP) for interventions [10]. Digital treatments in somatic healthcare services currently have limited reimbursement, with only one dedicated NCMP code generating minimal revenue. Consequently, the coding for the digital treatment program relies on other NCMP codes such as patient-reported outcome measures and participation in the initial video group session. Financial sustainability is only reached after treating around 1,700 patients. For the digital program, patients pay a £30 fee for the initial video session, but no OPPs is charged for the digital treatment program. For regular face-to-face consultations in specialised somatic healthcare, an OPP equal to £30 per appointment is charged.

### Description of participants

Of the 23 identified stakeholders, 19 agreed to participate in the study. The reasons for not participating were that they had not implemented the digital treatment program (*n* = 1) or time constraints (*n* = 1), and some did not respond to the invitation or the follow-up email (*n* = 2). Interviews with three initially identified participants were not conducted as data saturation was reached; meaning no new information emerged in the final interviews that contributed significantly to addressing the research question [11]. The sample was therefore deemed both sufficient and information rich. Among the 16 final participants, 7 were males and 9 were females. The sample represented several implementation roles, as defined by the CFIR: High-level Leader (*n* = 1), Mid-level Leaders (*n* = 4), Implementation Leads (*n* = 1), Implementation Team Members (*n* = 1), Other Implementation Support (*n* = 3), Innovation Deliverers (i.e. dietitians working with the clinical part of the patient flow) (*n* = 2) and Innovation Recipients (i.e. physicians referring patients to the treatment program, hereafter called referring physicians) (*n* = 4). The interviews lasted an average of 38 min (18–64 min).

### Coding and analysis

The analysis was performed stepwise with deductive thematic analysis, as described by Braun and Clarke [7, 12]. CT, HKH and RMFK were involved in this process. First, the audio files were transcribed by the automated transcription feature in Microsoft Word (Microsoft^®^ Word for Microsoft 365 MSO (Version 2408)). To ensure accuracy and completeness, two members of the research team (CT and HKH) independently listened to the audio files while carefully reviewing the transcripts. They manually corrected errors, clarified unclear passages, and ensured that all spoken content including pauses, nonverbal utterances, and relevant emotional cues was accurately captured. Second, the transcripts were uploaded to NVivo Version 12 and coded individually by two members of the research team (CT and HKH) using a deductive approach guided by the Consolidated Framework for Implementation Research (CFIR) codebook. This codebook includes predefined constructs and subconstructs that capture domains relevant to implementation processes. After all transcripts were coded individually, they were compared and merged into one file per interview by CT and HKH. Discrepancies between the initial coding were discussed with RMFK. Third, the files were reviewed to identify overarching themes by CT and HKH, and ratings were assigned to determine valence (i.e. the direction of the influence on the implementation process) and strength (i.e. the magnitude of the influence on the implementation process) according to predefined criteria through a consensus process. The valence rating was adapted from the CFIR Rating Rules by Damschroder et al. [13], and assessed whether the construct had a negative, positive or neutral influence and to what degree. According to this systematic coding approach, CT and HKH assigned a numerical score to each construct for each respondent before pooling the ratings together. The valence was either + (positive influence), - (negative influence) or 0 (neutral influence), while the strength was either 2 (major influence) or 1 (minor influence). The strength was determined by assessing the level of degree of consensus among participants, intensity of language used, and inclusion of concrete examples [13] (additional file 3). Finally, CT, HKH and RMFK discussed and organised the material into a final structure.

### Ethics

The study fell outside the scope of the Health Research Act [1] and could be conducted without the approval of the regional ethics committee. The study was registered in Helse Bergen’s internal control system for research, innovation and quality improvement system with project number 5226—5226 and received approval from the local Data Protection Officer who reviewed the study and the materials. Written informed consent was obtained from each participant after the purpose of the study, how the data would be handled and their rights as participants were explained. All identifiable information was removed from the transcripts to ensure that privacy was maintained.

### Reflexivity

The authors of this article are a multidisciplinary group of researchers and clinicians with a broad background and diverse focus areas. CT is a registered dietitian with extensive clinical experience working with IBS patients and has been involved in several projects, notably during the initial phase of the digital treatment program. HKH is a nurse with several years of clinical experience and has served as a research assistant in prior studies utilising CFIR and qualitative research methods. EKS is a gastroenterologist trainee with both clinical and scientific experience from the field of IBS. She also held the position of managing director of the digital treatment program for a brief period. LASV is a registered dietitian with both clinical and scientific experience, and currently held the position as head of the Section of Clinical Nutrition at HUH. GAL is a gastroenterologist at with extensive clinical and scientific experience from the field of IBS. She is also a professor and currently held the position as the leader of the Center for Nutrition at the University of Bergen. BB is a neuroscientist with a wide-ranging scientific experience and is the managing director of the National Competence Centre for Functional Gastrointestinal Diseases. She also developed and serves as the managing director of the digital treatment program, but she derives no financial benefits from it. In addition to this, she is also an associate professor and head of the BrainGut Research Group at the University of Bergen. RMFK is a clinical psychologist with a broad background in implementation science and is the leader of the work package for implementation science at the Norwegian Research Centre for digital mental health services. Some of the co-authors were included as participants and interviewed in this study. These co-authors were involved in early-stage idea generation and informal discussions prior to the initiation of the study, but did not influence the design or methodological decisions. To maintain analytical integrity, these co-authors were not involved in the data analysis or interpretation processes, and only contributed during the final writing phase of this article. Only three authors (CT, HKH, and RKFM) were involved in the development of the interview guide, data collection, data analysis, interpretation and writing of the results. CT collaborates closely with some study participants as part of an ongoing quantitative randomised controlled trial investigating the clinical effects of the digital treatment program. This dual role may introduce some personal and interpersonal reflexivity, as her prior relation to some of the participants could influence interpretation and analysis. Furthermore, this dual role might have influenced the data collection process by shaping the dynamics of the interviews. In contrast, HKH had no prior relationship with the participants and had a key role in conducting the interviews, analysing and interpreting the results. While CT may have had preconceived ideas about barriers and facilitators in the implementation process due to her concurrent research, neither HKH nor RMFK held such preconceptions. Their involvement in the analysis helped ensure a more balanced interpretation of the data. Reflexivity was actively addressed through regular team discussions to ensure a balanced and transparent analysis process.

## Results

The 48 CFIR constructs assessed in this study include nine constructs within the subdomain Role in the Individuals Domain. While the roles identified as applicable to the project are documented and integrated into the overall presentation of the findings, they are not presented separately in the following sections. Of the remaining 39 CFIR constructs assessed in this study, 31 were perceived as determinants relevant to the implementation of the digital treatment program (additional file 4). In the following sections, we present the results in the context of the five CFIR domains and their related constructs.

### Innovation domain

Table 1 shows the ratings assigned to identified CFIR constructs within the Innovation Domain and examples of quotes supporting the rating. The *innovation sourc*e facilitated implementation, as participants trusted the group that developed the educational content and the digital treatment. The long-standing tradition of treating gastrointestinal disorders at HUH also supported referrals. While some participants valued the program’s *evidence base*, there was debate about its strength. The program’s *relative advantage* was seen as a facilitator, though one referring physician felt it was no better than usual care. The program’s *adaptability* to routine care was viewed positively, but *complexity* was a barrier due to the numerous steps involved for both deliverers and referring physician. The lack of a clear definition of the innovation added to this *complexity*. While *design* and *cost* were mentioned, they weren’t decisive. The high costs related to the development of the digital treatment program were noted, though unclear as a barrier.

**Table 1:** Ratings assigned to identified CFIR constructs in the innovation domain

Construct	Rating	Example quote
Innovation source	+2	*“I think that has affected a lot, right. That this (irritable bowel syndrome) has been a focus at Haukeland since, well ever since, I would say, in the 90s, right.” (referring physician, participant 14)*
		*“It is the largest professional community for different gastrointestinal disorders in the country. Um (pause) so you can think that people feel a stronger connection to it (the digital treatment program), that is, it’s more quality ensured, and you know something about the professional community.” (referring physician, participant 11)*
Innovation evidence-base	0 (mixed)	*“The engagement from the dietitians and gastroenterologists, as well as the work being done regarding that, and having it presented several times with regards to the program, it seems to be a fantastic program. But that is not synonymous with the quality that a patient or a patient group will experience and that must be examined and then we’ll have to wait and see.” (high-level leader, participant 4)*
Innovation relative advantage	>+2	*“IBS is a patient group with limited healthcare services. I mean, they have some services, but they don’t truly meet their actual needs.” (high-level leader, participant 4)) *
		*“I do have a few things that could be relevant to discuss with the patients, but I don’t think that referring them (to the digital treatment program) would be very beneficial.” (referring physician, participant 11)*
Innovation Adaptability	+1	*“I think we can see the outlines of a promising patient pathway, more precise one consultation for your IBS to get a diagnose and some medical advice, and then a patient education pathway before you go back to your general practitioner.” (mid-level leader, participant 10)*
Innovation Complexity	-1	*“We shouldn’t make it so huge that those who work with it can’t handle it.” (mid-level leader, participant 10)*
		*“I realize now that I didn’t know much. Uhm, I did know a little about what it was about, but it’s only when you’ve worked with it for a while that you really understand how extensive it (the digital treatment program) is.” (implementation deliver, participant 2)*
		*“The digital treatment program is a multidisciplinary treatment service, but now it’s only clinical dietitians that answers the questions. And we do receive quite a few questions meant for doctors, physiotherapists and psychologists there. Yes, we find that it’s a bit challenging, especially regarding the grey areas - when to respond or not.” (implementation deliver, participant 1)*
Innovation Design	0 (not conclusive)	*“Yes, both the digital platform and the content. It’s true that we could have hired acters to read in these videos and made it more user friendly that way. We could have had experts on digital education in the project so that you could create something that has been shown to be effective in a different way, a better way than what we have done. It’s always a lot you could have done better.” (implementation leads, participant 5)*
Innovation Cost	0 (not conclusive)	*“The costs have been very high.” (implementation leads, participant 5)*

### Outer setting domain

Table 2 shows the ratings assigned to identified CFIR constructs within the Outer Setting Domain and examples of quotes supporting the rating. The COVID-19 pandemic was a *critical incident* that facilitated the implementation. The *attitudes* within the clinical research practices acted as barriers, while the supportive professional community facilitated the implementation. Existing *partnerships & connections* facilitated the implementation, although a lack of government initiatives created barriers. Issues related to *policies & laws* posed as barriers, with regulatory issues and a “siloed” organisation of the Norwegian healthcare system. However, professional development and national guidelines supported the implementation. The funding system was inadequate for transitioning the program from research to routine care, making *financing* as a barrier.

**Table 2:** Ratings assigned to identified CFIR constructs and subconstructs in the outer setting domain

Construct	Rating	Example quote
Critical incidents	+2	*“The road was a bit like, a crash, because I mean we had start to work with it (the digital treatment program), but then you got Covid, and then you had to digitalize the physical patient course.” (mid-level leader, participant 10)*
Local Attitudes	0 (mixed)	*“It also concerns the incentives related to recognition and career development in clinical research. […] And then you have the cultural barriers, and there are many. I think the most important one is the culture among patients. They are used to see their doctor in person.” (implementation leads, participant 8)*
		*“They have supported it (the digital treatment program) as a professional environment, like a community. I haven’t heard a single gastroenterologist speak negatively about the digital treatment program.” (high-level leader, participant 4)*
Partnerships & Connections	0 (mixed)	*“That’s what is unique about this, that it was a national competence centre. Therefore, you had a network and therefore you had a lot of external relations and therefore you got a lot of relations that you could leverage to develop this and that could cheer for you and write support letters and so on.” (implementation leads, participant 8)*
		*“In January 2016, the directory for eHealth was started then, we approached them and met three working on eHealth and asked about their plans for digital treatment in the future and they didn’t have anything in the pipeline. […] So, they couldn’t send us in any directions on which type of digital platform we should go for.” (implementation leads, participant 5)*
Policies & Laws	-1	*“It all starts with who has the right to health care, right. Because it’s clear that sometime, with this type of treatment programs, you will maybe cross over to the responsibility of the primary care. And that’s not what we in the specialist care should do. We should stick to our responsibilities and use our resources, as we have more than enough to utilize our resources on. So, we cannot start doing things that are not for our purpose, even though research projects and innovation projects often take a broader approach because it is entirely natural for them to have a wider perspective. However, when we now face this regarding patient treatment, there is a harder reality concerning who holds the responsibility. Therefore, you do not want to encroach on the responsibility area of primary healthcare, and you also do not want to encourage over-treatment. And everyone agrees that this is not very wise with regards to the patients, because this is patients that typically are in follow-up at the primary care at some times and at the specialist care at some times. The fact that we can’t support them in a unified manner digitally also when it’s beyond the responsibility of the specialist care, that’s unfortunate.” (mid-level leader, participant 13)*
		*“We have been forced to set it (the coding) up based on the infrastructure that already exists for outpatient care, and that is not adapted for digital treatment.” (implementation leads, participant 5)*
		*«What might have been an obstacle is (pause) security and data protection. That always applies and there are strict demands that hinders us in achieving good development, and it is a quality assurance, just to be clear, so it is an important factor, but it’s always a fight between data protection, IT-security and they who wants to produce and make new innovations.” (other implementation support, participant 6)*
		*«And then you can talk about the professional innovations that has been within the area, namely the increased significance of diet for IBS. That is a professional development that has taken place over the last ten years […]. And the placement of patient education as a part, a key element in managing the problem, that’s also something that helps.” (mid-level leader, participant 10)*
Financing	-2	*“I think that there are some practical issues, such as the fact that we didn’t have clear guidance from the Directorate of Health regarding financing and how we should earn money or secure the financing of the digital treatment program. That wasn’t clear when we transitioned into clinical practice. […] But at the same time, we have managed despite all the uncertainty, right? So, it is not impossible to start, but you need to have support because it is a risk. Um, so that’s one thing, and then the coding and finances, and in a way, um, when it (the digital treatment program) doesn’t quite fit the coding system, that is certainly a barrier.” (mid-level leader, participant 9)*
Societal pressure	+1	*“One thing is that there is a lot of focus in the media and everywhere you see, there is a lot of focus on gastrointestinal disorders.” (other implementation support, participant 7)*
Market pressure	+1	*“We have been early adopters on digital treatment and self-management which means the providers market has not been mature enough, but now there are some more providers that increases the maturity.” (mid-level leader, participant 15)*
Performance-Measurement Pressure	+1	*“[…] it’s expected that we should digitalize, now it’s an emphasis on reduction in waiting time, and waiting lists should be eliminated – we cannot take in patients unnecessarily. So, the expectations from the health government and others are quite strong.” (other implementation support, participant 7)*

### Inner setting domain

Table 3 shows the ratings assigned to identified CFIR constructs within the Inner Setting Domain and examples of quotes supporting the rating. Poor integration of the *information technology infrastructure* was identified as a barrier, while the *work infrastructure* facilitated the implementation. The lack of *relational connections* between experts on digital treatment in the somatic healthcare system was a barrier, while the collaboration among clinics facilitated the implementation. A high *tension for change* facilitated the implementation because previous attempts were proven too resource demanding. A *compatibility* with existing workflows facilitated the implementation. However, the digital platform was not compatible with digital treatment making this a barrier. The *relative priority* construct reflected lower priority for IBS patients as a barrier, yet the digital program was prioritised over existing treatment options, facilitating implementation. The program was *aligned with the overall mission*, facilitating implementation. Although the internal *funding* was available, considerable effort was required along the way. The *physical space* available to the implementation team and deliverers facilitated the implementation. The lack of *access to knowledge* on implementation of digital treatment in the somatic healthcare system posed as barriers to implementation, while the establishment of protocols for delivery during the implementation process facilitated the process.

**Table 3:** Ratings assigned to identified CFIR constructs and subconstructs in the inner setting domain

Construct	Rating	Example quote
Information Technology Infrastructure	-1	*“And then there’s (sighs) all these digital systems that need to communicate with each other.” (implementation leads, participant 5)*
		*“It’s easy to talk about the technology. In a way it’s both. It’s a prerequisite for realizing a digital service, yet a lot of time has been spent facilitating the technology. And when the solution has been developed in (name of technological platform), it has required a lot of resources and has not been without problems. One can certainly say that the result is good, but it’s been a long journey.” (mid-level leader, participant 13)*
Work Infrastructure	+1	*“One aspect is the structure of this, namely that both the department of gastroenterology and the department of clinical nutrition is under the same clinic. That’s an important prerequisite for making this happen.” (mid-level leader, participant 10)*
Relational Connections	0 (mixed)	*“So, it’s about finding the right people to talk to and finding someone who can make decisions. That has been very challenging, so I decided that if we are to make any progress at all, we need to choose our own solution.” (implementation leads, participant 5)*
		*«In my experience, there has been an amazing culture between the eHealth and the clinic, and the collaborators have been engaged, they have been motivated and not at least creative. I could never have managed without them to achieve as much success as we have had with the digital treatment program. So that collaboration is priceless.” (other implementation support, participant 6)*
Communications	-1	*“I feel that I’ve received very little information along the way.” (other implementation support, participant 7) *
		*“It seems that those working with the technology themselves are not clear about the plan for the technology, how they envision its use, and how it should be developed.” (mid-level leader, participant 13)*
Culture Recipient-Centeredness	+1	*“We are a community with several people interested in functional gastrointestinal diseases and we have a long tradition for it and acceptance for it. So, I would almost believe that we are more positive towards this patient group and that we have a long tradition of researching and working clinically with them. I think this tradition might be what makes things easier here, although it could have worked other places.” (referring physician, participant 12)*
Culture Learning-Centeredness	+1	*“And the organization has patience with that (innovations) and understand that this type of changes requires time and resources. I believe that this has definitely contributed to its flourishing.” (other implementation support, participant 7)*
Tension for Change	+2	*“We’ve been responsible for the physical courses for many years. We tried a specialized outpatient clinic for IBS patients for a while, but we found it to be too extensive. And the main reason why it became too extensive was that the significant need for information and interdisciplinarity.” (mid-level leader, participant 10)*
Compatibility	0 (mixed)	*“The digital treatment program is established on a different technological platform than our other digital treatment programs. That’s unfortunate. […] This means that in terms of further development, they are now on a different platform that what is mainstream, which means that the development will be prioritized on the other platform.” (mid-level leader, participant 13)*
		*“It allows me to skip a lot of points in my usual conversation with the patient (laughs) because I know, that ‘Ok, now I refer the patient to the digital treatment program, and I don’t need to go through all the details that I would normally do because I know it will be taken care of’.” (referring physician, participant 12)*
		*“What is positive is the asynchronous communication. This means that healthcare personnel do not have to be (pause) on alert to respond immediately. Instead, they can regulate it by answering, for example, once a week. This enables the healthcare professionals to organize their own daily routines.” (other implementation support, participant 6)*
Relative Priority	0 (mixed)	*“Prioritizing this patient group, which is positioned quite low on the prioritization guidelines, against all other patients that needs help in the specialist health care system, that was, in many ways, what was on the wrong side of the scare when it comes to making a decision where the scale tips toward yes or no.” (implementation leads, participant 8)*
		*“So, this came up as an alternative that I as a professional could say, ‘Ok, I think this is a reasonable approach’.” (mid-level leader, participant 10)*
Mission Alignment	+1	*“And it has been timely to seek for digitalisation of treatment, so it’s been pressure from (name of a high-level leader) to establish such projects.” (mid-level leader, participant 10)*
Funding	-1	*“You’re met with positive vibes, but if we’ve asked for a single penny, there wouldn’t been anything out of the digital treatment program.” (high-level leader, participant 4)*
Space	+1	*“I think it’s (physical space) is very important because we have daily chats and discussions about how to solve problems.” (implementation deliver, participant 2)*
		*“It’s very fruitful that you are very solution-oriented and that you distribute tasks among yourselves and have a very good collaboration. It works like you work very well together.” (other implementation support, participant 3)*
Access to Knowledge & Information	0 (mixed)	*“There was no one that had the competence that came and could help with that type of (sighs) (pause) help or anything like that.” (implementation leads, participant 5)*
		*“They have developed good routines. So now, when we have new personnel, it’s easy to provide the training that’s needed.” (other implementation support, participant 7)*

### Individuals domain– characteristics

Table 4 shows the ratings assigned to identified CFIR constructs within the Individuals Subdomain of Characteristics and examples of quotes supporting the rating. High-level leaders were *capable* to fulfil their role and facilitating the implementation. Both mid-level leaders and referring physicians had an *opportunity* to fulfil their role and facilitating the implementation. Characterised by *motivation*, the deliverers, implementation leads, and other implementation support aided the implementation.

**Table 4:** Ratings assigned to identified CFIR characteristics in the individuals domain

Construct	Rating	Example quote
Capability	+2	*“We have this (high-level leader) who is very much (slams hand on table) ‘This is how it’s going to happen’ (laughs) and this can be very helpful in this context […]. It’s not every place that have such a decisive (name of a high-level leader) that believes in the concept.” (other implementation support, participant 7)*
Opportunity	+2	*“My role has been to say yes to the good ideas.” (mid-level leader, participant 10)*
		*“I have referred, both myself and referred back to the general practitioner with a recommendation to refer to the digital treatment program.” (referring physician, participant 16)*
Motivation	+2	*“Those who have worked with the digital treatment program are very innovation. And development-oriented people, so they find it very exciting to be part of these journeys. They really do.” (mid-level leader, participant 15)*

### Implementation process domain

Table 5 shows the ratings assigned to identified CFIR constructs within Implementation Process Domain and examples of quotes supporting the rating. The *teaming* facilitated the implementation through a trust-based group dynamic. Concerns regarding the *needs of deliverers* working digitally were raised, yet it was not demonstrated that these concerns were addressed during the implementation process. For referring physicians, the program allowed for their *need* for an available treatment option aiding the implementation, yet unclear referral routines and a lack of information on patient progress created barriers. Inadequate *planning* was a barrier to implementation due to unclear roles and responsibilities, as well as predefined steps and milestones. Some of the delivers reported the lack of *engaging* as a barrier, while others experienced ownership, which facilitated implementation. The *engagement of referring physicians and patients* was deemed important, though poorly addressed during the implementation. While the importance of incremental *doing* was valued, there was debated whether this had been adequately applied.

**Table 5:** Ratings assigned to identified CFIR constructs and subconstructs in the implementation process domain

Construct	Rating	Example quote
Teaming	+2	*“It’s very fun to work multidisciplinary (laughing). […] It’s just gold, you know. Good dialogues and good discussions. It’s incredible what comes up in settings like those. And where that setting is actually safe. That’s very important. That’s often the setting, we’re stressed, we’re different, but here we have this calm setting where there is no stress. That brings out the best in us.” (other implementation support, participant 6)*
Assessing needs Innovation delivers	0 (not conclusive)	*“Then we must hear from the people working with it (the digital treatment program) […]. That they feel that they feel they are doing a proper job and that they are true professionals, even though they are working digitally.” (mid-level leader, participant 9)*
Assessing needs Innovation recipients	0 (mixed)	*“Many clinicians feel like it’s very difficult with IBS patients, probably because they feel inadequate. And I think that the more tools we provide to clinicians, the more positively they will respond to the patients.” (referring physician, participant 12)*
		*“At first, I was told that only general practitioners could refer patients […] We tried to find those criteria online to see if only a general practitioner could refer or not. We became a bit uncertain, as we couldn’t find that out clearly, whether it had to be a general practitioner.” (referring physician, participant 16)*
		*“Well, I don’t really know more than that I refer patients, and then I hope the patients will join the course […]. I don’t really know how it works, actually. Does everyone get personal follow-up?” (referring physician, participant 14)*
		*“We also have to consider that it should be good for the patients, it has to be as good as the physical course, and it looks like it is, but we need to continue to keep an eye on it.” (mid-level leader, participant 9)*
Planning	-2	*“It (the transition to routine care) just happened, and we in the front line just had to manage it without having a plan in place.” (implementation deliver, participant 1)*
		*“A lot of issues and challenges have arisen along the way. […] That hasn’t been in place.” (other implementation support, participant 3)*
		*“We were suddenly told that we had a quarterly deadline related to coding, and we had two weeks to get it done.” (implementation deliver, participant 1) “Yes, and we really work to keep this deadline, and then it turns out that we’ve had one week wrong. We don’t even get any thanks for it; it’s just 'this has to be done' and then 'oh.'” (implementation deliver, participant 2)*
		*“Too many research projects operate on their own without a clear thought on how to transition from research and innovation into clinical practice.” (mid-level leader, participant 13)*
Engaging Innovation delivers	0 (mixed)	*“It’s about being seen and heard. […] You want to feel that decisions aren’t just being made above you.” (implementation deliver, participant 2)*
		*“It has been very exciting to be a part of the process with all the involved parties and see that we make things happen, even if there’s been all these bumps along the way.” (implementation deliver, participant 1)*
		*“It’s been a challenge where we’re being pushed regarding resources […] I find it a bit challenging how to balance it when you want that everyone to have some ownership and be informed, and (pause) at the same time ensure (pause) efficient operational activities.” (implementation deliver, participant 1)*
Engaging Innovation recipients	0 (not conclusive)	*“So, I believe that user involvement should have been integrated much earlier with the digital treatment program. But it’s not too late, so I think it should be a focus area moving forward.” (other implementation support, participant 6)*
Doing	0 (mixed)	*«So, there was a bit of a crisis, instead of us being prepared […]. That overarching framework has been lacking, where you gather everyone and sit down […] It has felt a bit rushed, as if they thought it would sort itself out.” (other implementation support, participant 7)*
		*“I think some of the smartest things […], we’ve taken a slow approach, so it’s been manageable, and we have been able to gain good experiences all along. I haven't just rushed in and said that now we are going to reach these and those goals, but we’ve taken our time, and that’s been incredibly valuable.” (implementation leads, participant 7)*

## Discussion

This qualitative study aimed to systematically investigate the important determinants in the regional implementation process of a digital treatment program into routine care in Norway using the CFIR. Using valence ratings to identify facilitators and barriers, five key facilitators and two key barriers were identified. The key facilitators included a trustworthy and knowledgeable innovation source, a high relative advantage of the digital treatment program over standard care, COVID-19 as a critical incident, a high demand for a change in the follow-up of IBS patients and an implementation team with a strong sense of safety and team spirit. Additionally, key characteristics among the individuals involved in the implementation were found to facilitate the implementation process: knowledgeable and confident high-level leaders, mid-level leaders and referring physicians with high levels of autonomy and power, and an implementation team and implementation deliverers with high levels of motivation and commitment. To ensure a seamless transition from research to routine care and the sustainable implementation of innovative digital treatment programs as routine treatment in secondary healthcare services, it is important to identify, acknowledge and utilise these facilitating factors. Key barriers included funding systems that were not facilitated for the transition from research to routine care and the lack of a predefined implementation plan. While not identified as key barriers, the complexity and undefined nature of the innovation, the organisation of the Norwegian healthcare system, regulations and laws, lack of political decisiveness, lack of interoperability between technological systems and uncertainty among the referring physicians were found to negatively influence the implementation process. Based on our systematic evaluation of the factors influencing the implementation process of a digital treatment program for patients with IBS in routine care, we have identified several key learning points derived from our interpretation of the findings.

### Assess readiness for change and ensure broad involvement from start

Among the key facilitators identified in our study, significant trust in the healthcare professionals responsible for the digital treatment program was profound. The referring physicians noted that they referred their patients solely based on their trust, even though they had limited knowledge about the digital treatment program itself. The aspect of a trustworthy innovation source is crucial, as this increases uptake among referring physicians and potentially patients as well. Limited research is available on the implementation of digital treatment programs for patients with IBS. However, for Internet-delivered Cognitive Behavioural Therapy, previous studies have shown that scepticism from other healthcare professionals acts as a barrier to both referrals and recognition from the professional community [14]. These contrary results may be related to other constructs we identified as facilitators, particularly the relative advantage. Based on our findings, we propose that the high demand for accessible and effective treatment options for patients with IBS and their low availability may influence referring physicians to be more positive and inclined to adopt digital treatment programs. The novelty of the digital treatment program and poor standard care for patients with IBS contributed to its perceived relative advantage, facilitating the process of promoting it to stakeholders, referring physicians and patients [15]. Although a high relative advantage and high innovation source trust were identified, it is important to note the barriers identified under the construct of assessing needs of referring physicians. The need for information and details about referrals were not properly assessed in the implementation plan, nor was the need for feedback on how the patients were doing. The necessity of understanding and including the needs of referring healthcare professionals has previously been underscored and aligns with our findings [16, 17]. It has previously been proposed to include referring healthcare professionals in the process of development to enhance the implementation process [17]. As we identified the referring physicians in our study to be characterised by autonomy and empowerment, they may serve as facilitators if they perceive tangible advantages and have sufficient competence about the referral process and the program itself. Zanni et al. [18] found similar results when they assessed the perception of healthcare professionals in referring cancer patients to a digital treatment program with cognitive behavioural therapy for insomnia. Taken together, our findings align with previous research and highlight the need for further research on how referring healthcare professionals’ attitudes toward digital treatment are influenced by the available treatment options and the early identification of their needs. Furthermore, our findings emphasise the importance of leveraging trustworthy innovation sources and a high relative advantage in future implementation initiatives.

### Facilitate collaborative engagement

Several factors related to teaming were identified as facilitators. Notably, the strong and supportive team spirit within and between the implementation team and the implementation deliverers was profound. A supportive and safe team environment is essential to foster open communication and collaboration and have previously been found to be associated with better implementation outcomes [19]. Our finding may be related to the characteristics of the individuals involved, as we identified significant motivation and championing behaviour among the implementation team and the implementation deliverers. They were highly committed, enthusiastic and believed in the digital treatment program. This was important, especially when challenges were met. This led to creative solutions, engagement and people taking an active part in the implementation process. In addition to collaborative engagement, development and integration in an existing medical clinic were identified as facilitators. Explicit support from decisive high-level leaders and autonomous mid-level leaders was important both for integration in the clinic and for fostering engagement and ownership. Our findings align with previous research in which leadership engagement and organisational location within larger specialised organisations were identified as facilitators [14, 20]. Another factor that may influence the nourishing team spirit identified in our study is the physical space that was available to the implementation team and the implementation deliverers. The importance of physical co-location in the delivery and implementation of digital treatment aligns with previous research [14]. This is important, as there is diversity in the organisation of digital treatment programs, with both regional and national services operating. Taken together, our findings highlight the importance of identifying and facilitating collaborative engagement during the planning and implementation of digital treatment from all parties involved, starting with the planning of the research project.

### Early establishment of a comprehensive implementation plan

The lack of a predefined implementation for the transition from research to routine care was identified as a major barrier. This became evident in multiple domains. Under the Implementation Process Domain, the uncertainty about roles and responsibilities, timelines and milestones of the implementation process resulted in frustration and confusion among most of the key stakeholders. Our finding of the lack of an implementation plan as a barrier to implementation is well known, and it was previously proposed that the plan should be comprehensive to ensure that all potential factors are identified at an early stage [20, 21]. Early identification of potential barriers could have prevented some of the frustration and confusion and highlighted the barriers related to the outer setting at an earlier stage. Although the barriers in the outer setting are well known, including the lack of government initiatives, silo-based healthcare systems, funding systems and reimbursement systems that are not facilitated for digital treatment programs, early identification of how these factors affect the implementation process is important [20]. It is important to highlight these barriers to navigate health policy pushes and address the need for more flexible funding systems and ensure that healthcare resources are utilised effectively and that digital solutions are leveraged to promote more sustainable healthcare systems. Taken together, our findings add to the literature indicating that an early and comprehensive implementation plan is a prerequisite for effective and sustainable implementation.

### Strengths and limitations

Our study included participants from a broad range of individuals involved in the implementation process, thereby highlighting different perspectives. The use of the CFIR in both the design and development of the interview guide, coding and design, was crucial to the systematic identification and categorisation of the relevant factors. Another strength of our study lies in the involvement of several individuals during the entire process from study design to analysis, enhancing the objectivity and legitimacy of the results. This study focused specifically on the regional implementation process within the public healthcare system, targeting key stakeholders directly involved in that process. Accordingly, individuals from private partners, political decision-makers, and patients were not included, reflecting our deliberate choice to concentrate on those with direct implementation roles. Although perspectives from private collaborators and policy actors could offer additional insights into broader contextual factors, and patient experiences provide valuable information about intervention reception, these aspects were beyond the scope of this study. Notably, patient satisfaction with the digital treatment program has been described in a previous publication [6]. There may also be a risk of recall bias among the participants because the interviews were conducted several years after the initiation of the digital treatment program. However, the interviews were conducted shortly after the regional implementation phase, which was the primary focus of this study.

## Conclusion

By utilising the CFIR, we systematically identified barriers and facilitators in the regional implementation process of a digital treatment program for patients with IBS in routine care. Our findings highlight the complexity of the implementation process in the setting of a public healthcare system. Factors that are critical for the success of the implementation process are: a trustworthy innovation source, high relative advantage, a cohesive implementation team, and the characteristics of individuals involved, such as support from knowledgeable high-level leaders and autonomous mid-level leaders and motivated implementation team members and deliverers. Several modifiable factors are important to address early in implementation to increase the effectiveness of the process and ensure the sustainability of the innovation, most notably, a clear and iterative implementation plan and a funding system that is more facilitated to support innovations in the transition from research to routine care. Our results indicate that there is a critical need for enhanced focus, expertise and knowledge in implementation of digital treatment programs in somatic routine care, with implications that extend beyond IBS to other clinical domains.

## Supplementary Information

Supplementary Material 1.

Supplementary Material 2.

Supplementary Material 3.

Supplementary Material 4.

## Data Availability

The datasets presented in this article are not readily available because data generated, analysed, and reported during the current study are not publicly available due to it being potentially identifying, but are available in a slightly shortened, de-identified form from the corresponding author on reasonable request. Requests to access the datasets should be directed to the corresponding author.

## Abbreviations

IBS: Irritable bowel syndrome
CFIR: Consolidated framework for implementation research
HUH: Haukeland university hospital
RHA: Regional health authority
OPP: Out-of pocket payment
ISF: Innsatsstyrt finansiering (the Norwegian term and abbreviation for performance-based grant)
DRG: Diagnosis-related group
ICD-10: International classification of diseases
NCMP: Nordic classification of medical procedures
