# Supplementary material for: Implementing a digital treatment program for patients with irritable bowel syndrome into routine care: a qualitative evaluation of barriers and facilitators perceived by key stakeholders
Source: BMC Health Serv Res. 2025 Aug 9;25:1049. doi: 10.1186/s12913-025-13171-0 (PMC12335064; doi:10.1186/s12913-025-13171-0)
Supplement: Supplementary file 1 — Supplementary Material 1. [file 12913_2025_13171_MOESM1_ESM.docx]

**INTERVIEW GUIDE – IMPLEMENTATION OF THE DIGITAL TREATMENT PROGRAM IN THE WESTERN HEALTH REGION**

Aim:

What were the barriers and facilitators in the implementation of the digital treatment program for patients with irritable bowel syndrome (IBS) in the western health region? What have we learned that we can utilize in the further nationalisation process of the digital treatment program?

- Individually (administrative staff and leaders) / focus group (clinicians), semi-structured in-depth interview
- Duration: approximately 40-60 minutes
- To the interviewer: describe how the interview went/thoughts about the interview (did we gain anything from the interview, etc.) in notes to yourself

Format:

- The interview begins with a short and informal conversation.
- The interviewer explains the purpose of the interview, including clarification about anonymisation, confidentiality, and information about audio recording.
- The interviewer then guides the participant through a series of pre-formulated questions, with the possibility for follow-up questions as needed.
  - Remember to let the participant speak freely, but feel free to use follow-up questions if the conversation stalls.

Opening Question:

- Can you describe your role in the implementation of the digital treatment program?
  - What did you know about the digital treatment program before you got involved in the implementation?

*Once the informant(s) have finished discussing their role, you can move on.*

Questions about the implementation process:

"The theme of this interview is the factors that have hindered and promoted the implementation of the digital treatment program as a treatment option in the western health region. Can you tell us about your experiences during this process?"

*Let the informant(s) speak freely. Use the questions below if the conversation stalls. Remember not to give examples or leading/closed questions.*

- What promoted the work of implementing the digital treatment program?
- What hindered the work of implementing the digital treatment program?
- How could the implementation of the digital treatment program have been improved?
- What factors contributed to whether specific measures worked or did not work?
- How has the implementation of the digital treatment program affected your workday? *If the informant(s) do not speak spontaneously, you can ask the follow-up questions.*
  - How did the digital treatment program fit into the usual work routines?
  - Are you aware of whether the digital treatment program had to be adapted to fit into your/their normal work routines?
  - Are you aware of whether any work routines on your/their part had to be adjusted for the digital treatment program to fit in?
- How have you experienced any external factors, such as political, economic, or professional guidelines, have affected the implementation of the digital treatment program? *If the informant(s) do not speak spontaneously, you can ask the follow-up questions.*
  - How have these conditions hindered or promoted the introduction?
  - What type of external funding was necessary to introduce and deliver the innovation?
    - How accessible was this funding?
- How have you experienced any conditions within the western health region, such as location, culture, leadership support, and work environment, have affected the implementation of the digital treatment program? *If the informant(s) do not speak spontaneously, you can ask the follow-up questions.*
  - How have these conditions hindered or promoted the introduction?
  - How have you experienced that the digital treatment program has been prioritized in relation to other available treatment options?
  - Are you aware of whether other treatment options influenced the implementation of the digital treatment program?
  - What type of internal funding was necessary to introduce and deliver the innovation?
    - How accessible was this funding?

Once the points above have been discussed, you can ask for advice for future implementation with the next question:

"You have mentioned several barriers you have experienced during the implementation. How could these conditions have been improved?"

*When the informant(s) have finished discussing the conditions regarding implementation, you can move on.*

Questions about the innovation:

"Now that we have talked a little about the implementation, I would like to discuss the digital treatment program itself."

- What was your perception of digital treatment for irritable bowel syndrome before you got involved in the digital treatment program?
- How do you compare the digital treatment program with other existing treatment options for patients with IBS?
  - For those working directly with IBS patients:
    - What was it like to work with IBS patients before the digital treatment program became available?
    - What is it like to work with IBS patients after the digital treatment program has become available?
- How do you perceive the quality of content and design of the digital treatment program?
  - How do you think the quality has hindered/promoted the implementation?
- From a patient perspective:
  - How do you feel that the digital treatment program meets the needs of patients?
  - In what ways?
  - How do you think the digital treatment program will impact patients with IBS?
- From a healthcare provider perspective:
  - How do you feel that the digital treatment program meets the needs of healthcare professionals working

Closing:

“Before we wrap up, I wonder if there's anything else you think is important to mention that we haven’t discussed?”

- What do you believe is essential to have in place for a successful process when the digital treatment program becomes a national treatment option?
- I will briefly go through the key points I have noted to summarise the main points that have emerged.
  - Is there anything you would like to correct or add?

*“Thank you for taking the time to answer our questions, and if you think of anything afterward, please feel free to get in touch.”*
