# Supplementary material for: Implementing a digital treatment program for patients with irritable bowel syndrome into routine care: a qualitative evaluation of barriers and facilitators perceived by key stakeholders
Source: BMC Health Serv Res. 2025 Aug 9;25:1049. doi: 10.1186/s12913-025-13171-0 (PMC12335064; doi:10.1186/s12913-025-13171-0)
Supplement: Supplementary file 3 — Supplementary Material 3. [file 12913_2025_13171_MOESM3_ESM.docx]

Table 1 Criteria used to assign ratings to constructs and subconstructs. Adapted from Damschroder et al [13]

| Rating | Criteria |
| --- | --- |
| -2  -1  0  +1  +2 | The construct or sub-construct acted as a major barrier to the implementation of the digital treatment program in the western health region. Two or more participants provided direct examples on how the construct or sub-construct acted as a major barrier.  The construct or sub-construct acted as a barrier to the implementation of the digital treatment program in the western health region, but was only mentioned in general statements or briefly without direct examples.  The construct or sub-construct identified both barriers and facilitators, yet the overall impact on the implementation of the digital treatment program in the western health region was negative.  The construct or sub-construct identified both barriers and facilitators of equal magnitude in contradictory statements from credible participants, resulting in an overall neutral impact on the implementation of the digital treatment program in the western health region.  The construct or sub-construct was only mentioned in passing without valence, and no conclusions could be drawn about it’s impact on the implementation of the digital treatment program in the western health region.  The construct or sub-construct acted as a facilitator to the implementation of the digital treatment program in the western health region, but was only mentioned in general statements or briefly without direct examples.  The construct or sub-construct identified both barriers and facilitators, yet the overall impact on the implementation of the digital treatment program in the western health region was positive.  The construct or sub-construct acted as a major facilitator to the implementation of the digital treatment program in the western health region. Two or more participants provided direct examples on how the construct or sub-construct acted as a major facilitator. |
