# Supplementary material for: Implementing a digital treatment program for patients with irritable bowel syndrome into routine care: a qualitative evaluation of barriers and facilitators perceived by key stakeholders
Source: BMC Health Serv Res. 2025 Aug 9;25:1049. doi: 10.1186/s12913-025-13171-0 (PMC12335064; doi:10.1186/s12913-025-13171-0)
Supplement: Supplementary file 4 — Supplementary Material 4. [file 12913_2025_13171_MOESM4_ESM.docx]

Table 2 Definition of the CFIR domains, constructs and subconstructs identified in this study. Adapted from Damschroder et al [7]

| Domain | Construct and subconstruct | Definition (the degree to which) |
| --- | --- | --- |
| Innovation domain  (i.e. conditions related to the digital treatment program being implemented) | Innovation source  Innovation Evidence-Base  Innovation Relative advantage  Innovation Adaptability  Innovation Complexity  Innovation Design  Innovation Cost | The group that develops the digital treatment program is perceived to be trustworthy and knowledgeable.  There is strong evidence demonstrating the effectiveness of the digital treatment program.  The digital treatment program surpasses standard care.  The digital treatment program can be adapted to integrate well into existing patient pathways.  The digital treatment program consists of many steps and lacks a clear definition.  The technological platform and educational content of the digital treatment program are perceived to be well-designed.  The costs associated with the development of the innovation are affordable. |
| Outer setting  (i.e. conditions lying outside of the western health region) | Critical incidents  Local Attitudes  Partnerships & Connections  Policies & Laws  Financing  External pressure  Societal pressure  Market pressure  Performance-Measurement Pressure | Unexpected events support the implementation process and delivery of the digital treatment program.  Sociocultural traditions and values support the implementation process and delivery of the digital treatment program.  External entities are connected to the implementation team and the inner setting, thereby supporting the implementation process and delivery of the digital treatment program.  Political provisions, regulations, and guidelines support the implementation process and delivery of the digital treatment program.  Funding and reimbursement systems support the implementation process and delivery of the digital treatment program.  Mass media focus supports the implementation process and delivery of the digital treatment program.  Competition between external entities supports the implementation process and delivery of the digital treatment program.  Expectations and benchmarking metrics support the implementation process and delivery of the digital treatment program. |
| Inner setting  (i.e. conditions related to the western health region) | Structural Characteristics  Information Technology Infrastructure  Work Infrastructure  Relational Connections  Communications  Culture  Recipient-Centeredness  Learning-Centeredness  Tension for Change  Compatibility  Relative Priority  Mission Alignment  Available Resources  Funding  Space  Access to Knowledge & Information | Technological systems are available in the western health region to support the implementation and delivery of the digital treatment program.  The structural organisation of the inner setting supports the implementation and delivery of the digital treatment program.  Internal networks and teams within the western health region support the implementation and delivery of the digital treatment program.  The information sharing practices in the western health region support the implementation and delivery of the digital treatment program.  Shared values around caring for IBS patients exist in the western health region that support the implementation and delivery of the digital treatment program.  Shared values around continuous learning exists in the western health region that support the implementation and delivery of the digital treatment program.  A collective feeling exists that a change is needed for IBS treatment.  The digital treatment program integrates well with the work routines in the western health region.  The digital treatment program is prioritised over other treatment options.  Implementing and delivering the digital treatment program aligns with the broader objectives and aims in the western health region.  Financial resources are available from the western health region to implement and deliver the digital treatment program.  Physical facilities are available from the western health region to implement and deliver the digital treatment program.  Competence and guidance are available in the western health region to implement and deliver the digital treatment program. |
| Characteristics of the individuals involved | Capability  Opportunity  Motivation | The individual(s) has the knowledge, decisiveness, and confidence needed to effectively carry out their role.  The individual(s) has the authority and resources to effectively carry out their role.  The individual(s) has the commitment and motivation to effectively carry out their role. |
| Implementation process domain (i.e. conditions related to the actions and plans carried out during the implementation initiative) | Teaming  Assessing needs  Innovation deliverers  Innovation recipients  Planning  Engaging  Innovation deliverers  Innovation recipients  Doing | The team spirit creates a fruitful ground to effectively implement the digital treatment program.  The needs of the deliverers are systematically gathered and utilized to inform the implementation and delivery of the digital treatment program.  The needs of the recipients (healthcare workers/referees and IBS patients) are systematically gathered and utilized to inform the implementation and delivery of the digital treatment program.  A comprehensive and iterative implementation plan is employed before implementation of the digital treatment program.  The deliverers are invited to engage in the implementation of the digital treatment program.  The recipients (healthcare workers/referees and IBS patients) are invited to engage in the implementation of the digital treatment program.  The implementation of the digital treatment program is performed stepwise. |
